# Ferroptosis and inflammation are modulated by the NFIL3-ACSL4 axis in sepsis associated-acute kidney injury

**DOI:** 10.1038/s41420-024-02113-0

**Published:** 2024-08-04

**Authors:** Zhong Xiao, Jie Zhang, Zhimin Qiu, Hongbing Liu, Hua Ding, Hi Li, Yuanxin Liu, Xiaohua Zou, Juan Long

**Affiliations:** 1 Department of Anesthesiology, The Affiliated Hospital of Guizhou Medical University, Guiyang, China; 2 College of Anesthesiology, Guizhou Medical University, Guiyang, China; 3 Department of Critical Care Medicine, Shaoxing People’s Hospital, Shaoxing, China; 4 Emergency Department, The First Affiliated Hospital of Wenzhou Medical University, Wenzhou, China; 5 Emergency Department, The First People’s Hospital of Xiaoshan District, Hangzhou, China; 6 Department of Breast Surgery, The Affiliated Hospital of Guizhou Medical University, Guiyang, China

**Keywords:** Pathogenesis, Molecular biology

## Abstract

Sepsis-associated acute kidney injury (SA-AKI) increases the risk of death in patients with sepsis, and its major pathological change is the death of renal tubular cells. However, the mechanism of its occurrence remains unclear. Sepsis can lead to circadian dysregulation, and the rhythm gene NFIL3 has been reported to regulate lipid metabolism. There is compelling evidence that has demonstrated that lipid peroxidation can cause cellular ferroptosis. In this study, we established the in vitro and in vivo models of SA-AKI and confirmed the presence of ferroptosis of the renal tubular epithelial cells in SA-AKI. In addition, analysis of the GEO database showed that NFIL3 was highly expressed in sepsis patients and was highly correlated with the key molecule of ferroptosis, ACSL4. The in vitro and in vivo data suggested that NFIL3 was involved in ferroptosis and inflammation in SA-AKI. Subsequently, loss-of-function experiments revealed that NFIL3 knockdown attenuated ferroptosis and inflammation in renal tubular epithelial cells by downregulating ACSL4 expression, thus protecting SA-AKI. In conclusion, this study is the first to illustrate the involvement of the rhythm gene NFIL3 in SA-AKI, providing new insights and potential therapeutic targets for SA-AKI.

## Introduction

Sepsis is the dysregulated host response to infection and can cause life-threatening organ failure [1]. The kidney is one of the most vulnerable target organs during sepsis, and can result in sepsis-associated acute kidney injury (SA-AKI), which accounts for more than 50% of AKI cases in the ICU and increases the risk of chronic kidney disease and death [2–7]. Despite current advances in medical technology and intensive care units (ICU), up to two-thirds of patients with sepsis or septic shock will develop SA-AKI [6]. Currently, there is still no effective treatment for it. SA-AKI increases the in-hospital mortality rate by 6–8 times, and contributes to a quarter of renal replacement therapy cases [8, 9]. Therefore, a better understanding of the mechanism of SA-AKI is critical to developing effective therapeutic strategies.

Renal tubular cell death is considered to be one of primary pathogenic events in SA-AKI. Regulated cell death includes focal death, cell necrosis, necroptosis, and ferroptosis [10, 11]. Ferroptosis, unlike other types of regulated cell death, recognized as a non-apoptotic cell death caused by iron-dependent lipid peroxide accumulation [12]. It has been revealed that ferroptosis plays a crucial role in diseases such as cisplatin-induced AKI, ischemia-reperfusion-induced AKI, and diabetic nephropathy [13–15]. A recent study reported that ferroptosis is involved in renal tubular epithelial cells during SA-AKI, and inhibition of ferroptosis attenuates septic kidney injury [16]. Therefore, ferroptosis may become an emerging therapeutic target for SA-AKI.

Sepsis can cause circadian rhythm dysregulation, which leads to the immune disorders and aggravates organ damage [17]. The circadian clock system comprises the primary clock genes, including CLOCK, ARNTL, PER, and CRY, and circadian rhythm effectors, including REV-ERBα, retinoic acid receptor-associated orphan receptors, and downstream clock-controlled genes. Disruption of circadian rhythms are associated with many diseases, including chronic obstructive pulmonary disease, cancer, and obstructive sleep apnea syndrome [18]. Recently, it has been found that the rhythm gene ARNTL is involved in ferroptosis [19]. Nuclear factor interleukin-3 (NFIL3) is also one of the rhythm genes with broad role in regulating development of immune cells [20], affecting T cell functions [21], and in processes such as liver metabolism and adipogenesis [22, 23]. However, the role of the rhythm gene NFIL3 in ferroptosis during sepsis has not been reported yet.

In this study, we analyzed the GSE134347 dataset and found that the rhythm gene NFIL3 is highly expressed in sepsis patients, inducing ferroptosis in renal tubular cells through upregulation of ACSL4. This work is the first to explore the role of the rhythm gene NFIL3 in SA-AKI, which deepened the understanding of the mechanism of SA-AKI and provided the basis for the therapeutic strategy in SA-AKI.

## Results

### Ferroptosis is involved in acute kidney injury in the CLP-induced mice

**Fig. 1:**
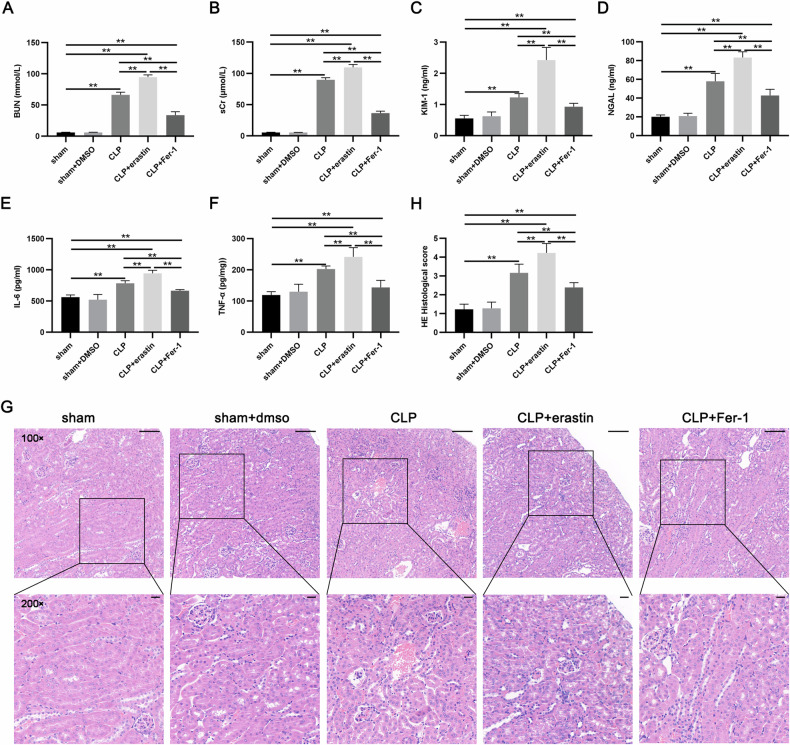
Ferroptosis is involved in CLP-induced AKI. Serum levels of (**A**) BUN, (**B**) sCr, (**C**) KIM-1, and (**D**) NGAL in the mice were measured. **E**, **F** The levels of IL-6 and TNF-α were examined in renal tissue by ELISA. **G**, **H** Representative HE staining pictures (100×, 200×) and renal tubular injury scores for each group of mice. Scale bars = 100 µm or 20 µm. Data represent mean ± SD from at least three independent experiments, *p < 0.05, **p < 0.01.

In SA-AKI, we investigated the impact of erastin and Fer-1 treatment to validate the role of ferroptosis. The levels of BUN, sCr, KIM-1, and NGAL in the serum were examined to reflect renal function, among which KIM-1 and NGAL are indicators of renal tubular injury, and NGAL is a sensitive biomarker for early AKI diagnosis [16, 24]. As described in Fig. 1A–D, erastin intervention further exacerbated CLP-induced kidney injury, while administration of Fer-1 alleviated septic kidney damage, as indicated by evidence from BUN, sCr, KIM-1, and NGAL. The pathogenic process of SA-AKI also involves an inflammatory response. Thus, to verify the occurrence of renal injury, we monitored the levels of inflammatory factors including IL-6 and TNF-α (Fig. 1E, F). Moreover, HE staining of the kidney indicated that sepsis caused tubular dilatation, cell detachment, tubular interstitial edema, and tubular brushlike-border disruption, and administration of eratin resulted in more serious pathological alterations. Fer-1 treatment, however, mitigated CLP-induced pathological damage (Fig. 1G, H). These results demonstrated that the ferroptosis had a role in the pathological development of SA-AKI.

**Fig. 2:**
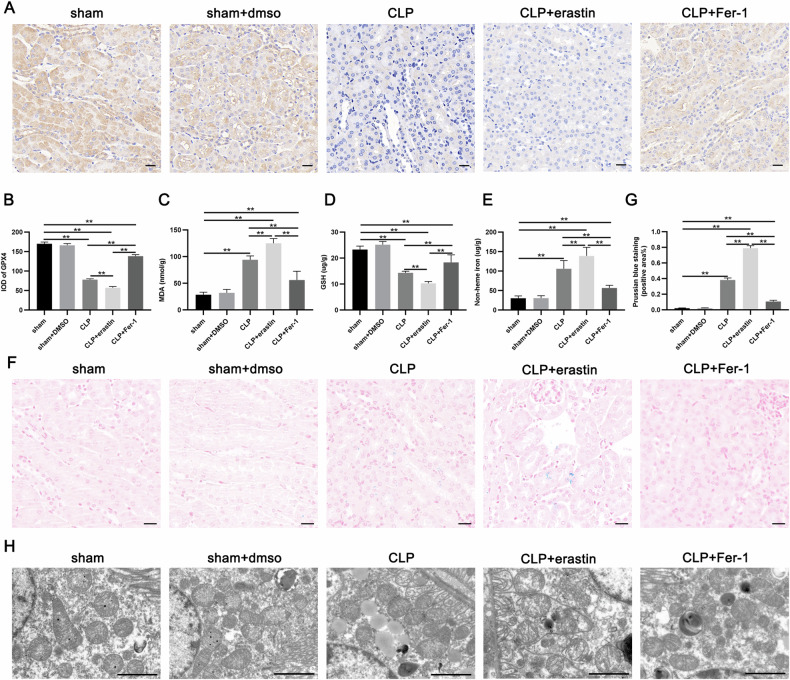
Ferroptosis is involved in SA-AKI. **A**, **B** Representative immunohistochemical images and quantitative analysis of GPX4 expression in renal tissue. Scale bars = 20 µm. **C**–**E** The level of MDA, GSH, and non-heme iron end products in renal tissue. **F**, **G** Representative images and quantitative analysis of Prussian blue staining. Scale bars = 20 µm. **H** Representative images of transmission electron microscopy (TEM) for each group. Scale bars = 2 µm. Data represent mean ± SD from at least three independent experiments, *p < 0.05, **p < 0.01.

Subsequently, we further investigated the changes related to ferroptosis in SA-AKI. IHC results depicted that GPX4, a key molecule of ferroptosis, was considerably lower in the CLP group in comparison with the sham and sham + DMSO groups, and was further decreased by erastin treatment, while Fer-1 treatment ameliorated the sepsis-induced GPX4 reduction (Fig. 2A, B). In addition, MDA and non-heme iron end products were substantially increased in CLP-induced AKI, accompanied by a significant decrease in GSH (Fig. 2C–E). A considerable increased renal heme iron in renal after CLP was observed after Prussian blue staining (Fig. 2F, G). Transmission electron microscopy results showed that the renal tubular epithelial cells depicted shortened mitochondria, and the number of cristae was reduced or disappeared after CLP, which are typical mitochondrial alterations in ferroptosis (Fig. 2H). The typical changes of ferroptosis in the renal after CLP were much more pronounced when intervening with erastin, whereas these changes were ameliorated when Fer-1 was administered beforehand (Fig. 2A–H). These data suggest the presence of ferroptosis in renal tubular epithelial cells during SA-AKI.

### LPS induces ferroptosis in HK-2 cells in vitro

**Fig. 3:**
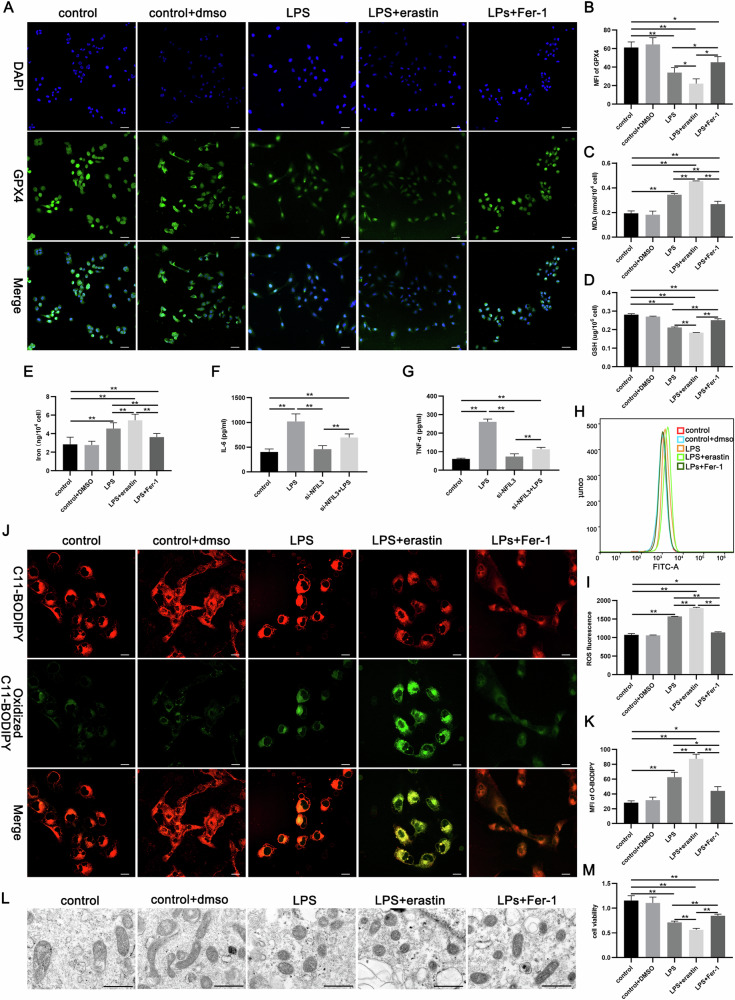
LPS induced ferroptosis in HK-2 cells. **A**, **B** Representative immunofluorescence images of GPX4 and quantitative analysis. Scale bars = 50 µm. **C**–**E** Quantitative analysis of MDA, GSH, and non-heme iron end products in each group of cells. **F**, **G** The levels of IL-6, TNF-α were examined in supernatant by ELISA. **H**–**K** ROS, lipid peroxidation levels, and their quantitative analysis. Scale bars = 50 µm. **L** Representative images of TEM in each group of cells. Scale bars = 500 nm. **M** The cell viability in each group detected by CCK8 assay. Data represent mean ± SD from at least three independent experiments, *p < 0.05, **p < 0.01.

Ferroptosis was observed in renal tubular epithelial cells during SA-AKI in CLP mice. Therefore, LPS-treated HK-2 cells could be used in an in vitro study. In line with the results of animal experiments, significantly reduced GPX4 expression in HK-2 cells by LPS, which was further decreased by erastin treatment. However, administration of Fer-1 ameliorated the LPS-induced GPX4 reduction (Fig. 3A, B). In addition, LPS resulted in an increase in cellular MDA and non-heme iron, accompanied by decreased GSH, and erastin-treated further worsened these results, whereas Fer-1 treatment reduced MDA and non-heme iron levels and increased GSH (Fig. 3C–E). The inflammatory factors IL-6 and TNF indicated LPS-induced injury to HK2 cells. As shown in Fig. 3, LPS-exposure led to the occurrence of cellular inflammatory response, which was further worsened by administration of erastin, while Fer-1-treated alleviated the LPS-induced inflammatory response and decreased the expression of IL-6 and TNF-α (Fig. 3F, G). We then observed characteristic indicators of ferroptosis, including cellular lipid peroxidation levels and mitochondrial morphology. The results showed that LPS promoted intracellular ROS production and lipid peroxidation. Similarly, these changes were exacerbated by erastin and attenuated by Fer-1 (Fig. 3H–K). As for cellular ultrastructure, LPS-treated HK-2 cells induced ferroptosis-related changes in mitochondria that were worsened by erastin and ameliorated by Fer-1 (Fig. 3L). The CCK-8 assay also suggested that LPS impaired cell viability, and much more reduced LPS-induced cell viability by erastin and alleviated by Fer-1 after LPS (Fig. 3M). Thus, LPS was shown to induce ferroptosis in HK-2 cells, supporting that SA-AKI is associated with ferroptosis in renal tubular epithelial cells.

### Rhythm gene NFIL3 is in sepsis significantly positively correlated with ACSL4, a pivotal contributor and regulator of ferroptosis

**Fig. 4:**
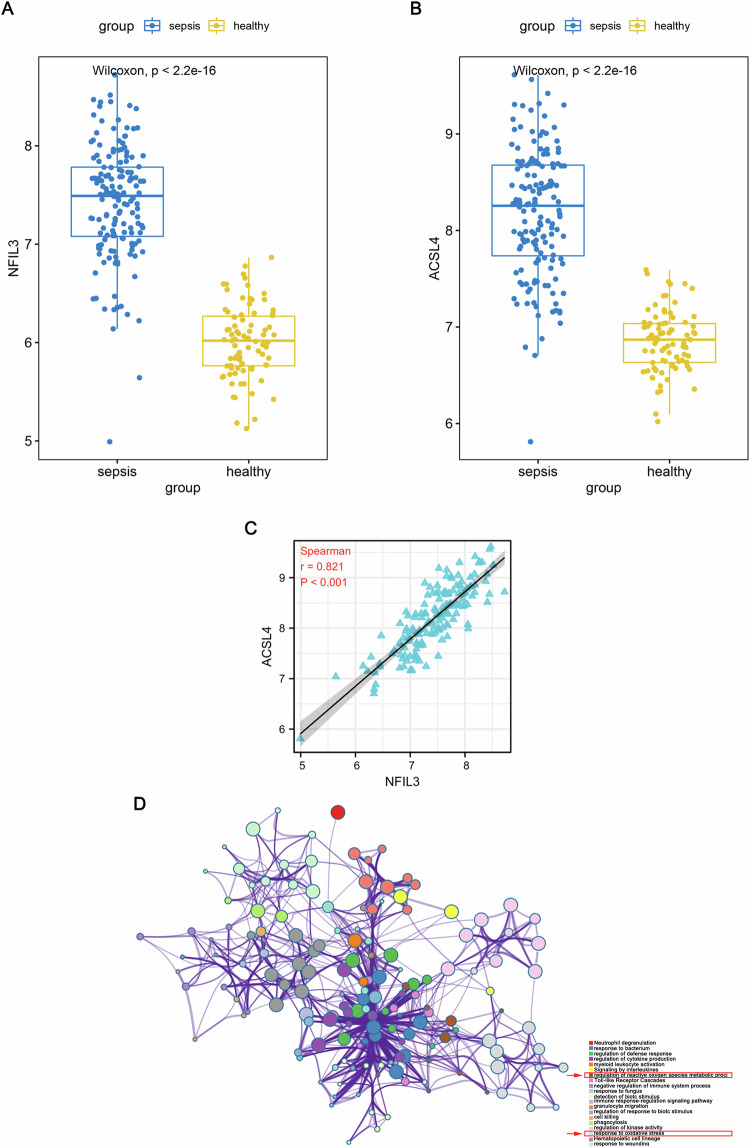
Rhythm gene NFIL3 in sepsis is significantly positively correlated with ACSL4. **A** NFIL3 mRNA expression in patients with sepsis and the healthy in GSE134347 dataset. **B** ACSL4 mRNA expression in patients with sepsis and the healthy in GSE134347 dataset. **C** Correlation between NFIL3 and ACSL4 in patients with sepsis in GSE134347 dataset. **D** The Gene Set Enrichment Analysis (GSEA) of differentially expressed genes between the septic and the healthy.

To explore the mechanisms causing ferroptosis in SA-AKI, we first analyzed the GSE134347 dataset and found that the rhythm gene NFIL3, together with important contributor to ferroptosis ACSL4, was significantly highly expressed in sepsis patients compared to healthy controls (Fig. 4A, B). What is noticeable is that the expression level of ACSL4, a key molecule in ferroptosis, was significantly and positively correlated with NFIL3 in sepsis patients (Fig. 4C). Based on the differentially expressed genes between patients with sepsis and normal control individuals, GSEA analysis was performed. The results showed that inflammation-related gene sets and oxidative stress related gene sets (Fig. 4D). Oxidative stress has been considered to contribute to lipid peroxidation, the key driver for ferroptosis. Taken together, our findings suggest that NFIL3 could be involved in ferroptosis of SA-AKI.

### High expression of the rhythm gene NFIL3 in sepsis upregulates the key molecule of ferroptosis, ACSL4

**Fig. 5:**
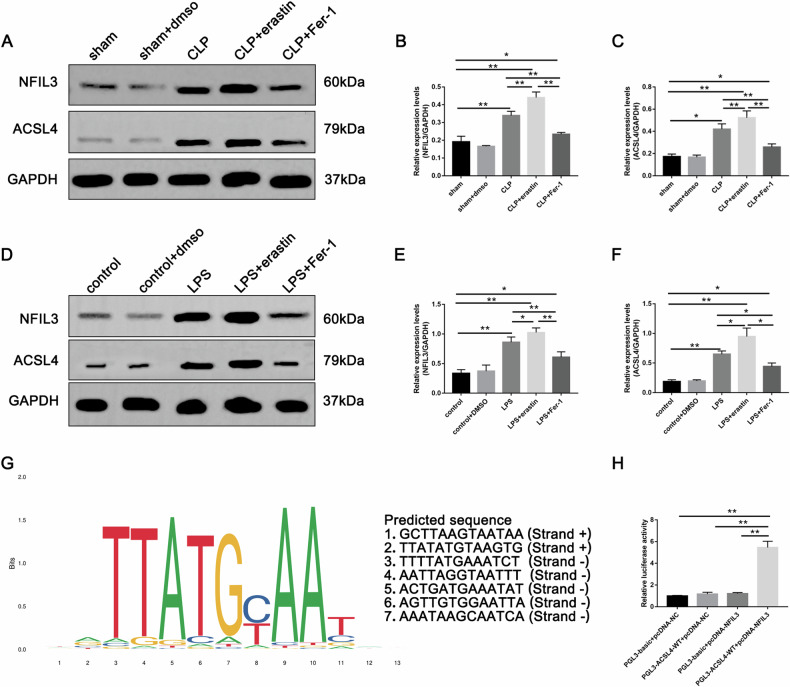
NFIL3 is involved in ferroptosis in SA-AKI. **A**–**C** The expression level of NFIL3 and ACSL4 in each group of mice. **D**–**F** Western blot analysis of NFIL3 and ACSL4 in HK-2 cells. **G** Predicted NFIL3-binding sites in the promoter region of ACSL4. **H** Luciferase assays were performed in 293T cell. Data represent mean ± SD from at least three independent experiments, *p < 0.05, **p < 0.01.

To determine whether ACSL4 mediated NFIL3 modulation is involved in ferroptosis of SA-AKI, we next verified the expression of NFIL3 and ACSL4 in the kidney for each group of mice using Western blot. In an agreement with analysis of GSE134347, renal NFIL3 and ACSL4 were upregulated in mice after CLP compared to sham and sham + DMSO groups. Erastin can further elevate renal NFIL3 and ACSL4 expression in septic mice, while Fer-1 inhibits their expression (Fig. 5A–C). Similarly, LPS-treated HK2 cells also had higher levels of NFIL3 and ACSL4 expression compared to the control. Their expressions were promoted by erastin, but decreased when pretreated with Fer-1 (Fig. 5D–F). Subsequently, by using the Jaspar database, we predict the binding sites of NFIL3 in the promoter region of ACSL4 and found seven putative binding sites (Fig. 5G). Therefore, we hypothesized that NFIL3 acts as a transcriptional activator of ACSL4. Next, a dual luciferase reporter assay was performed to determine the binding between NFIL3 and ACSL4. Here, we demonstrated that NFIL3 increased the gene expression of the ACSL4 (Fig. 5H). Collectively, it is suggested that NFIL3 is involved in the development of renal ferroptosis in SA-AKI and may be achieved through the regulation of ACSL4.

### NFIL3 mediates renal tubular epithelial cell ferroptosis and inflammation in SA-AKI through upregulation of ACSL4

**Fig. 6:**
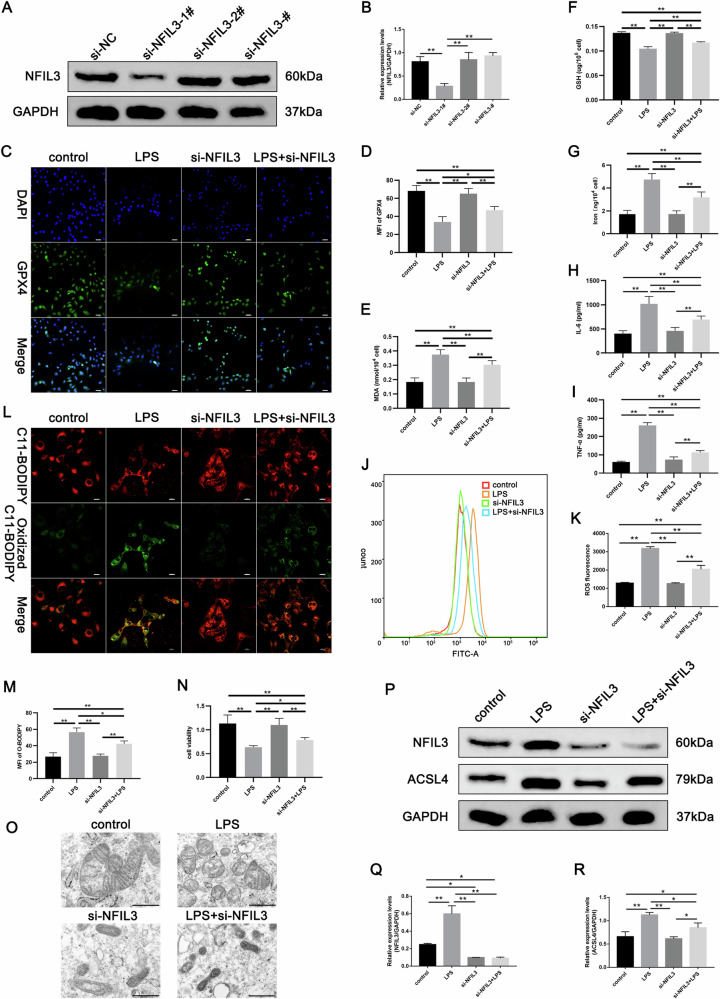
NFIL3 is involved in SA-AKI ferroptosis. **A**, **B** Western blot analysis of the knockdown efficiency of NFIL3 in HK-2 cells. **C**, **D** Representative images of GPX4 immunofluorescence and qualitative analysis of each group of cells after NFIL3 inhibition. Scale bars = 50 µm. Quantitative analysis of (**E**) MDA, (**F**) GSH, and (**G**) non-heme iron in each group. **H**, **I** Concentrations of IL-6, and TNF-α were examined in supernatant. **J**–**M** ROS, lipid peroxidation levels, and their quantitative analysis in each group. Scale bars = 50 µm. **N** The cell viability in each group detected by CCK8 assay. **O** Representative images of TEM. Scale bars = 500 nm. **P**–**R** Knockdown of NFIL3 followed by suppression of LPS-induced increase in ACSL4 expression. Western blot results of NFIL3 and ACSL4 expression in the indicated groups. Data represent mean ± SD from at least three independent experiments, *p < 0.05, **p < 0.01.

To verify whether NFIL3 causes ferroptosis in renal tubular epithelial cells in SA-AKI by regulating ACSL4, siRNA was utilized to knock down the expression level of NFIL3 in HK-2 cells. The knockdown efficiency was verified using Western blot. Due to highest efficiency, si-NFIL3-1# was used for subsequent experiments (Fig. 6A, B). The results of immunofluorescence indicated that knockdown of NFIL3 resulted in a considerable reduction in GPX4 expression (Fig. 6C, D). The changes in MDA, GSH, and non-heme iron in each group of cells were observed, and found that depleted NFIL3 expression decreased MDA and non-heme iron levels and increased GSH levels, similar to the after-treatment results of Fer-1 (Fig. 6E–G). At the same time, we detected the expression levels of IL-6 and TNF, and found that the inflammatory response was also changed with the knockdown of NFIL3 (Fig. 6H, I). In addition, the down-regulation of NFIL3 attenuated the elevated intracellular ROS levels and lipid peroxidation accumulation caused by LPS (Fig. 6J–M). The CCK-8 assay showed that downregulation of NFIL3 increased LPS-induced cell viability (Fig. 6N). The cellular ultrastructure also suggested that downregulation of NFIL3 ameliorated LPS-induced changes related to ferroptosis in cells (Fig. 6O). Additionally, the expression of ACSL4 was considerably reduced in the si-NFIL3 group (Fig. 6P–R). These findings demonstrate that inhibition of NFIL3 can attenuate LPS-induced ferroptosis and inflammatory response in renal tubular epithelial cells by downregulating ACSL4, which could have a protective effect against SA-AKI.

## Discussion

Previous studies have documented the association of SA-AKI with necrosis, apoptosis, and autophagy of renal tubular cell [25–27]. Recent researches have demonstrated that ferroptosis is a potential therapeutic target for SA-AKI [16, 28]. Consequently, we uncovered in this work that ferroptosis of renal tubular epithelial cells was produced during SA-AKI, and that NFIL3 may be a crucial node that exacerbates SA-AKI by promoting ferroptosis. We initially examined the data set to investigate the possible regulation mechanisms of SA-AKI. Sepsis patients showed a large upregulation of the ferroptosis-associated gene ACSL4, as well as a considerable upregulation of NFIL3. According to the analysis, we picked out the ferroptosis-related genes ACSL4 and NFIL3 for additional investigation.

Currently, a large number of laboratory studies have reported the role of ferroptosis in sepsis. Depletion of GSH, GPX4 inactivation, lipid peroxidation, and intracellular iron accumulation are the hallmarks of ferroptosis [29]. GPX4, as an antioxidant enzyme, can reduce the toxicity of lipid peroxides by increasing the activity of peroxidase, and is considered to be a major regulator of ferroptosis [30]. ACSL4, an enzyme involved in lipid metabolism, is another important participant in ferroptosis and serves as a crucial pro-ferroptosis gene, which can enhance the production of lipid peroxides and increase ferroptosis sensitivity [31]. Previous experiments have revealed that GPX4 expression decreases and ACSL4 expression increases during SA-AKI [28]. In this study, we obtained consistent results by testing GSH, lipid oxidation, iron levels, ACSL4, and GPX4 expression under SA-AKI or LPS-induced AKI conditions. At the same time, we also found that erastin or Fer-1 treatment can aggravate or alleviate SA-AKI, which further proves the involvement of ferroptosis in SA-AKI, and it is essential to explore its precise mechanism.

NFIL3, a basic leucine zipper transcription factor, is involved in the regulation of the biological clock, neuronal growth, and survival, as well as a regulatory role in innate and adaptive immunity [32–36]. In a study on circadian rhythms in patients with sepsis, it was suggested that sepsis suppresses the expression of the Circadian gene, such as CRY1, REV-ERBα, NR1D2, DBP, and PER2 [17]. REV-ERBα inhibition can regulate lipid metabolism by increasing fatty acid uptake and fat storage via upregulating of NFIL3 [21]. In addition, NFIL3 regulates hepatic lipid metabolism by inhibiting CREBH. It is well known that lipid peroxidation is recognized as the essential cause of ferroptosis [37]. In this study, our analysis based on the GEO database found that NFIL3 expression was elevated in patients with sepsis and significantly positively correlated with ACSL4. Consistently, in SA-AKI or LPS-induced AKI, renal tubular epithelial cells exhibited ferroptosis changes, accompanied by the same trend of increased expression of NFIL3 and ACSL4. Meanwhile, the ferroptosis agonist, erastin exacerbated renal injury and significantly upregulated NFIL3 and ACSL4 expression, whereas the ferroptosis inhibitor Fer-1 administration decreased NFIL3 and ACSL4 expression, thereby alleviating renal tubular epithelial cell ferroptosis and ameliorating renal injury. Therefore, we proposed that NFIL3 may be involved in developing ferroptosis in SA-AKI, and NFIL3 upregulates ACSL4 gene expression in transcription and promotes the occurrence of ferroptosis. According to the data analysis, the binding site of NFIL3 is located in the promoter region of ACSL4. We used promoter analysis, Western blot, and a dual luciferase reporter gene experiment to determine whether NFIL3 is a transcriptional activator for ACSL4, and briefly discuss the NFIL3-ACSL4 axis. Consistently, our laboratory results support the hypothesis that silencing NFIL3 can effectively inhibit ACSL4 expression and ferroptosis under conditions of SA-AKI and LPS-induced AKI. Our study showed that increased NFIL3 expression was followed by upregulation of ACSL4, but in NFIL3 depleted HK-2 cells, ACSL4 expression was notably reduced and cellular ROS, lipid peroxidation, inflammation, and altered mitochondrial ferroptosis were significantly improved. However, the precise binding site requires further study. Taken together the clue above, it is suggested that inhibition of NFIL3 can alleviate ferroptosis and inflammation by downregulating ACSL4. However, it still needs to be validated in animal models. To the best of our knowledge, our study is the first to identified the functions of NFIL3 and its association with ACSL4 in ferroptosis and inflammtion of SA-AKI, thereby, enhancing the understanding of mechanisms underlying SA-AKI. Importantly, this work may provide valuable clues to search novel therapeutic target for SA-AKI.

## Conclusions

In summary, ferroptosis may play an important role in cell death associated with SA-AKI. This study is the first to illustrate the involvement of the rhythm gene NFIL3 in SA-AKI and demonstrated that NFIL3 inhibition alleviates SA-AKI by suppressing ACSL4-regulated ferroptosis and inflammation, which may provide a new therapeutic strategy for SA-AKI.

## Materials and methods

### Ethics statement

All procedures in this study were conducted in accordance with the Animal Care Welfare Committee of Guizhou Medical University (Guiyang, China). The study also adhered to the relevant ARRIVE criteria and the National Institutes of Health guidelines outlined in their ‘Guide for the Care and Use of Laboratory Animals.’

### Experiments and animal models

Thirty male C57BL/6 mice (22–26 g, 8–10 weeks old) were purchased from the Zhejiang Vital River Experimental Animal Technology Co., Ltd. (Certificate No. SCXK (Zhe) 2019-0001, China). All mice were fed under SPF conditions with free access to water and food. They were randomly assigned to 5 groups: sham operation group (Sham group, n = 6), sham operation + DMSO group (Sham + DMSO group, n = 6), cecum ligation and puncture group (CLP group, n = 6), cecum ligation and puncture + Erastin group (CLP + E group, n = 6), and cecum ligation and puncture + Fer-1 group (CLP + F group, n = 6). All procedures in this study were carried out in compliance with the Animal Care Welfare Committee of Guizhou Medical University (Guiyang, China).

The mouse sepsis model was established by cecum ligation and puncture (CLP) according to the previous method [38]. Equal doses of DMSO and Erastin (20 mg/kg dissolved in DMSO) were given immediately postoperatively to the DMSO and CLP + E groups, respectively, and Fer-1 (5 mg/kg dissolved in DMSO) was given 2 h earlier to the CLP + F group. After 24 h, the mice were euthanized, and blood and tissues were collected for subsequent experiments. The animal grouping was known to the researchers without any blinding.

### Cell culture and treatment

Human proximal renal tubular epithelial cells (HK-2 cells) were purchased from the Shanghai Institute of Cell Biology. The cells were cultured in Dulbecco’s modified Eagle’s medium (DMEM) supplemented 10% FBS and 1% penicillin/streptomycin. Cells were treated with or without DMSO, LPS (200 μg/mL), Erastin (10 μM), or Fer-1 (1 μM), respectively, and were subsequently collected after 24 h for subsequent experiments. It was determined that none of the cells had Mycoplasma.

In addition, six-well plates inoculated with 5 × 10^4^ HK-2 cells/well were afterward cultured for 24 h. The cells were transfected with 50 nM NFIL3 siRNA. The siRNA was mixed with serum-free medium and transfection reagent and replaced with a complete medium after 6 h. After 48 h, proteins were extracted and transfection efficiency was assessed by Western blot assay.

### Enzyme-linked immunosorbent assay (ELISA)

The blood and renal tissue of the mice was collected. The levels of serum blood urea nitrogen (BUN) (boyun biotech, BYS0282), serum creatinine(sCr) (boyun biotech, BYS0209), Kidney injury molecule 1 (KIM-1) (solarbio, SEKM-0147), and neutrophil gelatinase-associated lipocalir (NGAL) (solarbio, SEKM-0119) were measured using the respective kits per the manufacturer’s directions. The levels of the IL-6 (proteintech. KE10007), and TNF-α(proteintech. KE10002) in the renal tissue were determined by standardized ELISA kits based on the manufacturer’s instructions.

### Detection of MDA, GSH, and non-heme iron in cell and kidney tissue samples

The collected fresh kidney tissues and HK-2 cells were assayed utilizing the malondialdehyde (MDA, solarbio, BC0025), glutathione (GSH, solarbio, BC1170), and tissue iron content assay (solarbio, BC5310) kits, respectively, and per the manufacturer’s instructions.

### Renal histopathology and immunohistochemistry (IHC)

Mouse kidney tissues were collected, instantly fixed in 10% neutral formalin for 24 h, paraffin-embedded, and cut into 4 μM slices. The hematoxylin-eosin (H&E) stained histopathological sections were examined under a light microscope (NIKON Eclipse Ci, Japan). The pathological changes in renal tissue were evaluated according to the morphology of renal tubular endothelial cells, the integrity of the brush border, the number of renal epithelial cells, and the condition of tubular necrotic cells [39]. The immunohistochemical sections were stained after incubation with GPX4 (Abcam, ab219592) antibody at 4 °C overnight. The integrated optical density (IOD) of GPX4 expression was quantified through an image analysis software, Image J, which is utilized for statistical analysis.

### Fluoroscopic electron microscopy

Fresh kidney tissues (about 1 mm × 1 mm × 1 mm) of the mice were collected and soaked in tubes containing electron microscopy fixative for 4 h. Subsequently, they were permeabilized, dehydrated, and embedded. Ultra-thin sections of tissues were made, and re-stained in uranyl acetate (30 min) and lead citrate (10 min). The changes in the samples were observed by transmission electron microscopy and images were collected.

### Prussian blue staining

The kidney tissues were fixed, dehydrated, embedded, and sectioned, the paraffin sections were dewaxed, and potassium ferrous hydroxide and hydrochloric acid were mixed in equal proportions into the Prussian blue staining solution. The sections were stained into the staining solution for 1 h, washed with distilled water, and the nuclei were re-stained with nuclear solid red, dehydrated, and sealed. The samples were then examined under a light microscope (NIKON Eclipse Ci, Japan), and Image J was used for image analysis.

### Western blotting

Briefly, after extraction of tissue or cellular proteins, electrophoresis was performed in 10% polyacrylamide gels and the isolated proteins were transferred to PVDF membranes (Millipore, Billerica, MA, USA). The cells were blocked for 2 h at room temperature in 5% BSA on a shaker and incubated with NFIL3 (cell signaling technology, 14312) or ACSL4 (affinity, DF12141) primary antibodies overnight in a 4 °C refrigerator. Secondary antibodies were incubated for 1 h at room temperature on a shaker and developed using the enhanced chemiluminescence reagent (Millipore). The protein bands were quantified through the Image J software.

### CCK8 assay

The cells were inoculated in 96-well plates at a density of 1 × 10^4^ cells/well in 100 μL of the medium, and the cell viability of different groups of cells was assayed using Cell Counting Kit-8 (MedChemExpress, HY-K0301) per the kit’s protocol. Ten μL of CCK-8 was injected into each well, and placed in the incubator for 2 h, then absorbance at 450 nm was quantified through an enzyme marker.

### ROS assay

ROS levels were detected by flow cytometry utilizing the 2,7-dichlorofluorescein diacetate (DCFH-DA) (Beyotime Biotechnology, S0033S). The dilution of DCFH-DA with a serum-free culture medium at 1:1000 was carried out until a final concentration of 10 µmol/L was reached. Afterward, the cell culture medium was removed by two-fold washing of the cells with serum-free medium, and the appropriate volume of diluted DCFH-DA was added. The cells were incubated for 20 min in a cell incubator at 37 °C and were washed thrice with the serum-free cell culture medium and collected afterward. The flow cytometer (488 nm excitation, 521 nm emission) was employed for the measurement of the fluorescence intensity.

### Lipid peroxidation assay

Cellular lipid peroxidation levels were detected through laser confocal microscopy using C11 BODIPY 581/591 (ABclonal, RM02821). Briefly, cells were washed twice with PBS per the manufacturer’s instructions, serum-free medium mixed with C11 BODIPY 581/591 (50 μM) was added, incubated for 1 h in a cell incubator at 37 °C, and washed twice with PBS to clear excess dye. The fluorescent signals were collected for analysis using a confocal microscope (Nikon, Japan) with excitation from 488 and 565 nm lasers, respectively.

### Bioinformatics analysis

The GSE134347 dataset was downloaded from the GEO database (http://www.ncbi.nlm.nih.gov/geo), a sample of 156 septic patients versus 83 normal individuals. The raw data CEL files were processed with the R language package, Oligo, and the Robust Multichip Analysis (RMA) method was utilized for subsequent analysis after standard normalization. The Limma package was employed for analyzing the differentially expressed genes between sepsis patients and controls, the genes whose|Log2 fold change| > 1 and P < 0.05 were defined as differentially expressed genes. The Metascape web tool completed the enrichment analysis.

### Immunofluorescence

The cells were washed thrice with PBS and the process was repeated twice while fixation in 4% paraformaldehyde occurred in between the two processes for 15 min. 1% triton was used for 10 min, followed by three washes with PBS and blocked with 5% BSA for 30 min. The primary antibody was used for GPX4 (Abcam, ab219592) overnight at 4 °C. The samples were washed thrice with PBS per and then incubated for 2 h with the secondary antibody, Alexa Fluor 488 (1:500) at room temperature, followed by staining of cells with 4′6-diamidino2-phenylindole (DAPI) (Solarbio, Beijing, China). The confocal microscope (Nikon, Japan) was utilized for observing the cells.

### Luciferase reporter assay

To assess ACSL4 gene promotor activity, 293T cells were seeded into 48-well plates and were co-transfected with ACSL4 luciferase reporter plasmid, pcDNA-NFIL3 and the Renilla control reporter serving as an internal control. The lysates were collected at 48 h after transfection, and luciferase activity was measured with the dual‐luciferase assay (Promega, E1910).

### Statistical analysis

All data analyses were performed by SPSS with Prism software. All data were presented with mean ± SD and analyzed using one-way ANOVA, and LSD was utilized for direct comparison between the two groups. The variations among the two groups were measured with the help of two-sided t-test. Correlation analysis was made by using Spearman correlation coefficient. Three-fold replication of all the experiments was performed, and all data analysis was considered statistically different with a P < 0.05.

### Supplementary information

SUPPLEMENTAL MATERIAL

## Data Availability

The raw data supporting in the study are included in the article. Further inquiries can be directed to the corresponding authors, without undue reservation.
