# Supplementary material for: Ferroptosis and inflammation are modulated by the NFIL3-ACSL4 axis in sepsis associated-acute kidney injury
Source: Cell Death Discov. 2024 Aug 4;10:349. doi: 10.1038/s41420-024-02113-0 (PMC11297963; doi:10.1038/s41420-024-02113-0)
Supplement: Supplementary file 1 — SUPPLEMENTAL MATERIAL [file 41420_2024_2113_MOESM1_ESM.pdf]

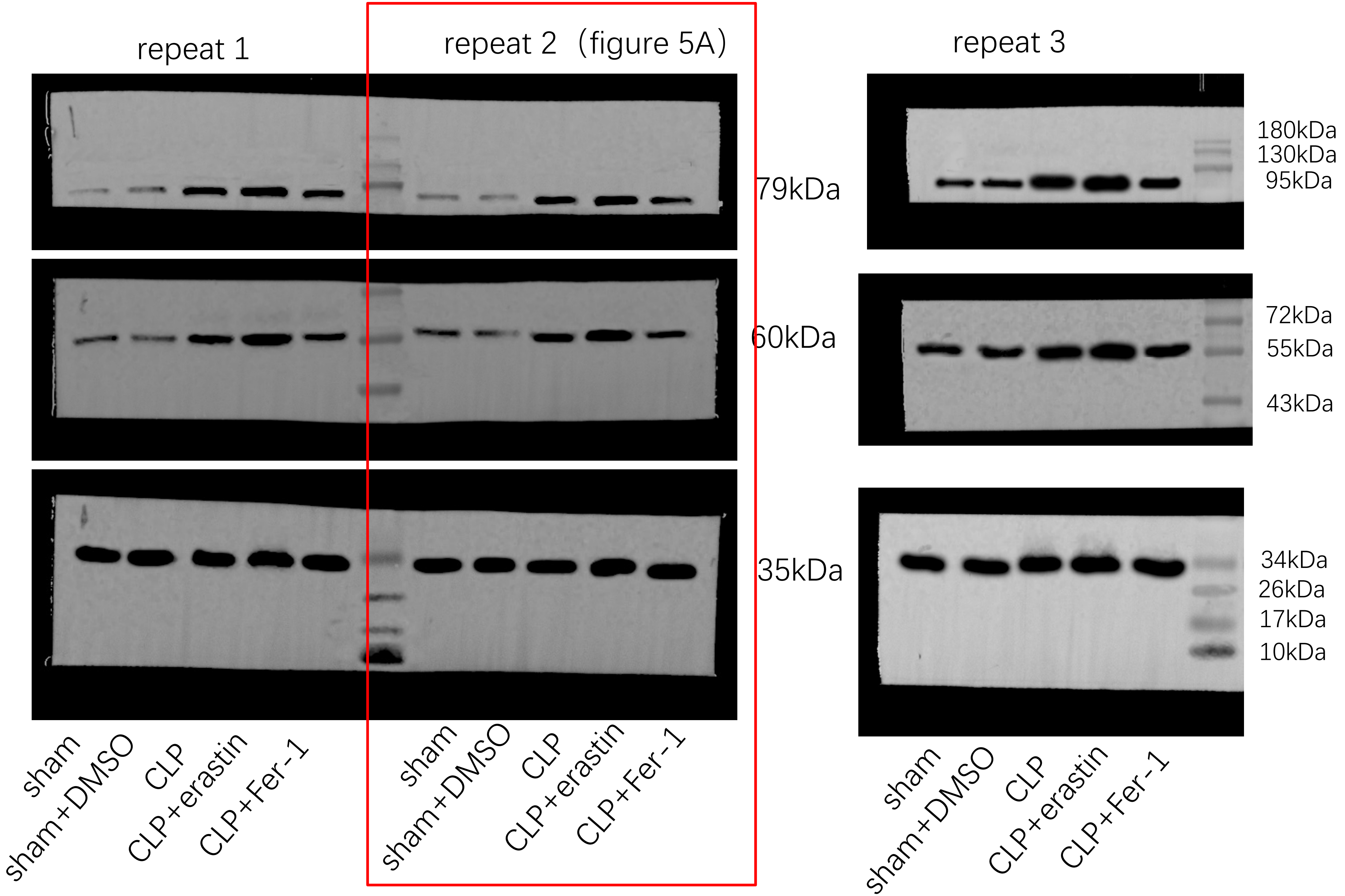
repeat 1
repeat 2 (figure 5A)
repeat 3
79kDa
60kDa
35kDa
180kDa
130kDa
95kDa
72kDa
55kDa
43kDa
34kDa
26kDa
17kDa
10kDa
sham
sham+DMSO
CLP
CLP+erastin
CLP+Fer-1

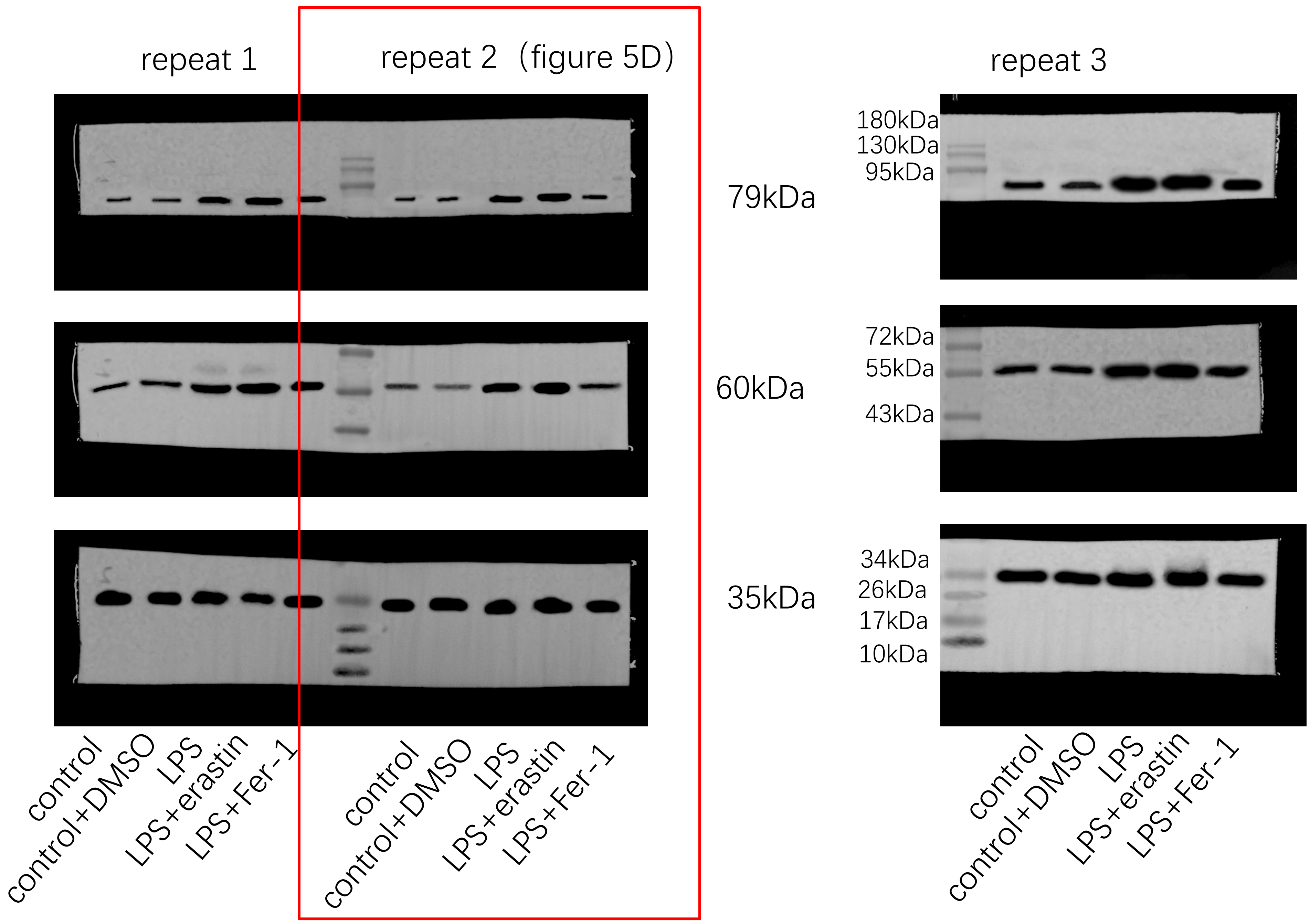
repeat 1
repeat 2 （figure 5D）
repeat 3
79kDa
60kDa
35kDa
180kDa
130kDa
95kDa
72kDa
55kDa
43kDa
34kDa
26kDa
17kDa
10kDa
control
control+DMSO
LPS
LPS+erastin
LPS+Fer-1
control
control+DMSO
LPS
LPS+erastin
LPS+Fer-1
control
control+DMSO
LPS
LPS+erastin
LPS+Fer-1

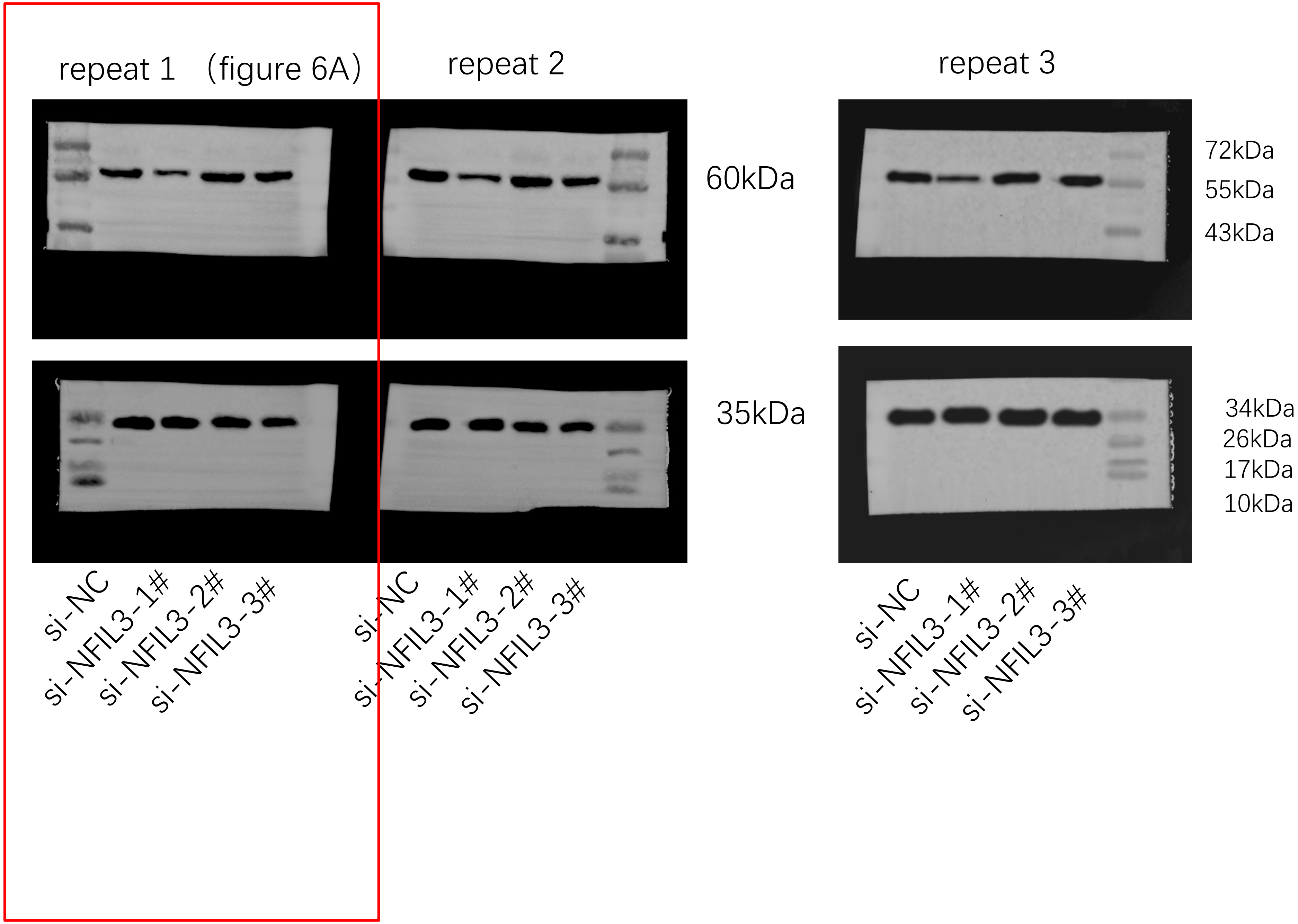
repeat 1 （figure 6A）
repeat 2
repeat 3
60kDa
35kDa
72kDa
55kDa
43kDa
34kDa
26kDa
17kDa
10kDa
si-NC
si-NFIL3-1#
si-NFIL3-2#
si-NFIL3-3#
si-NC
si-NFIL3-1#
si-NFIL3-2#
si-NFIL3-3#
si-NC
si-NFIL3-1#
si-NFIL3-2#
si-NFIL3-3#

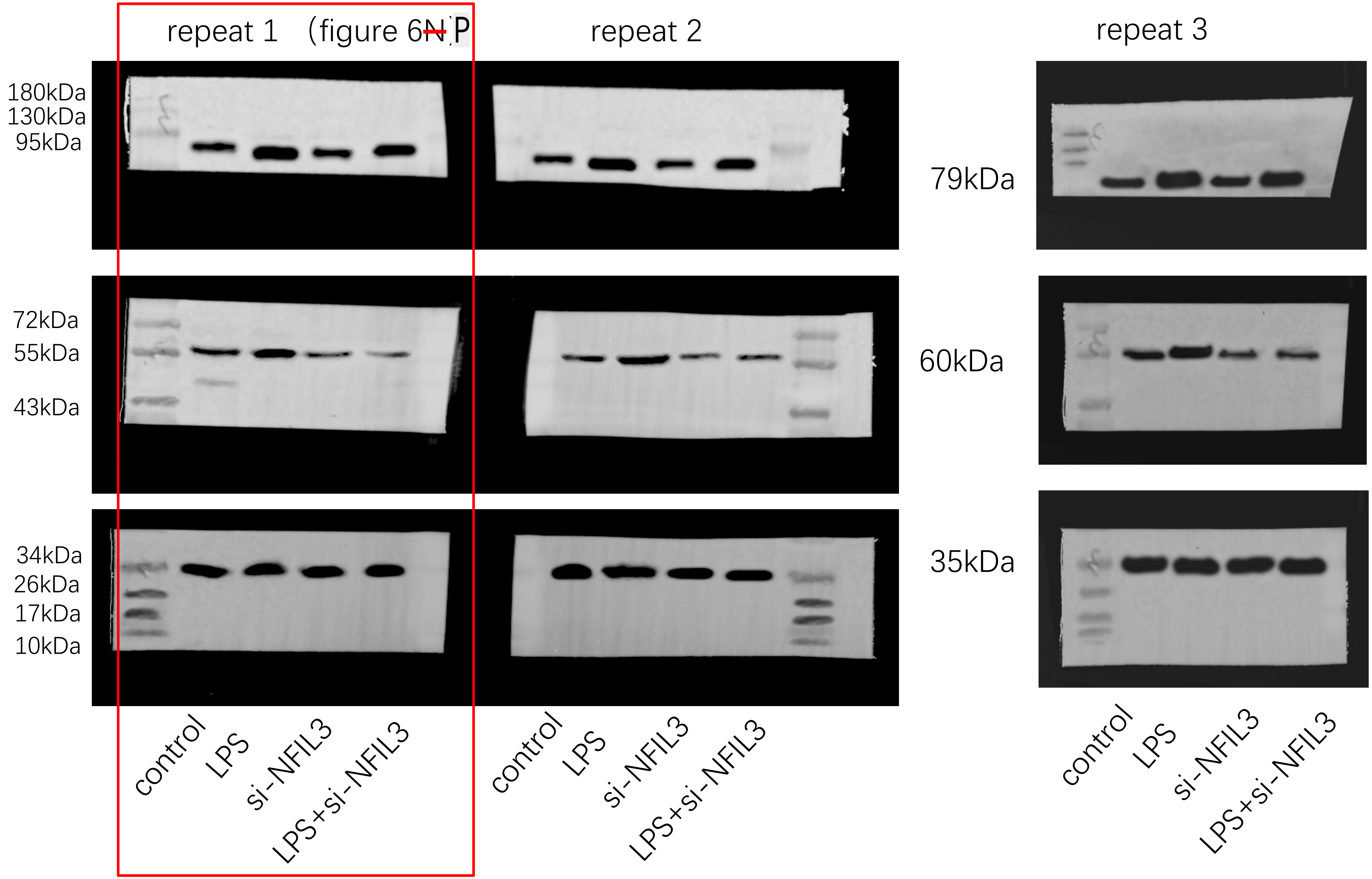

repeat 1 (figure 6~~N~~P
repeat 2
repeat 3
180kDa
130kDa
95kDa
79kDa
72kDa
55kDa
43kDa
60kDa
34kDa
26kDa
17kDa
10kDa
35kDa
control
LPS
si-NFIL3
LPS+si-NFIL3
control
LPS
si-NFIL3
LPS+si-NFIL3
control
LPS
si-NFIL3
LPS+si-NFIL3
